# A Clinical Lecture on Mitral Regurgitation

**Published:** 1888-02

**Authors:** Nathan S. Davis

**Affiliations:** Professor of the Principles and Practice of Medicine and Clinical Medicine in the Chicago Medical College, Chicago, Ill. Delivered at the Mercy Hospital.


### A CLINICAL LECTURE ON MITRAL REGURGITATION.

BY NATHAN S. DAVIS, M. D., LL. D.,

Professor of the Principles and Practice of Medicine and Clinical Medicine in the Chicago Medical College, Chicago, Ill. Delivered at the Mercy Hospital.

(Reported for the ATLANTA MEDICAL AND SURGICAL JOURNAL by Wm. Whitford, M. D.)

GENTLEMEN—The case I show you this morning is in some respects an interesting one. The history we get is rather imperfect, but from what we can learn the patient claims not to have been sick until about a month prior to his admission at the hospital. He is about 55 years of age and is employed on a railroad as switchman. He has a thin, wiry and muscular look about him, as you see. About a month ago, while carrying coal upstairs, his breath gave out, and he became so oppressed for breathing that he has been unable to take any active exercise since. Any attempt at exertion causes shortness of breath, and yet he acknowledges having no pain at any time. I do not think there is any elevation of temperature, but on examining him he presents such a complication of physical signs and appearances that some of them seem inexplicable on the supposition that there was nothing wrong with him until four or five weeks ago. The changes seem not at all likely to take place in that short period of time to such an extent as they are here manifest. His chest is not shrunken in any of its dimensions; it apparently maintains about the usual *contour* on both sides. If anything, the intercostal spaces in the lower part on both sides, particularly the left, are a little fuller than we would expect in a man in the same condition of flesh that he is. There is a noticeable bulging, or a little more fullness of the intercostal spaces on the left side than is usual, as though pressure was from within. The right side, as we look at him more closely, is also moderately filled, but not so much so as the left. His breathing is very short, and he wants to sit up in bed very nearly all the time so as to enable him to breathe more fully and easily. As we remove the wearing apparel from the front part of his chest, you will see it is fairly well preserved as regards *contour*. You observe, too, that the venous pulse in the neck is very active, and in applying percussion there is a moderate degree of pulmonary resonance anteriorly on both sides, not, of course, strictly natural. There is moderate clearness in the infraclavicular region; below the mammary region there is more or less dullness on both sides. On the right side it continues down, embracing the whole region of the liver. Dullness on the left side is not so marked. Posteriorly there is an increased area of dullness in the cardiac region; in fact, there is more dullness here than would be accounted for by an increase of space occupied by the heart or pericardium. There is a lack of intestinal resonance; the feet and legs are strikingly œdematous. In applying the ear to the chest we get a sharp, rather coarse râle just at the end of each inspiration; the inspirations are not deep. We get the idea at once, in listening, that he imperfectly inflates the lung. The inspirations are forced and a little short, and at the end of each inspiratory act we hear a sharp, bronchous, crackling sound, which we would sometimes call a subcrepitant râle—a sort of an intermediate sound between a dry and moist râle. It is limited to the end of each short inspiratory act, giving us the impression that while very much of the lung is under pressure, yet it is capable of being forcibly inflated. He has little or no cough, and apparently no expectoration, at least as far as we can ascertain now. His bowels are regular and urine normal.

Placing the stethoscope a little left of the sternum, directly over the cardiac space, close listening will detect the cardiac sounds, and gives us at once a regular to-and-fro murmur, showing that the systole brings one murmur and the diastole another, indicating that we have regurgitation on the left side. When we come to the right of the sternum, over the area where we would look for any fault in the tricuspid, we get another almost equally plain double murmur, showing that the tricuspid does not close the auriculo-ventricular opening, and that explains at once the free pulsation observed in the patient's neck. If you place the stethoscope over that point, you get a strong, free pulsation, which indicates that the man has some affection of the cardiac valves, a trouble that undoubtedly dates far back. It is highly probable he has been laboring for a long time under a moderate thickening and defect in the mitral valves—not to such an extent, however, as to prevent him from attending to his daily business, for he is a man whose external appearances are indicative of perseverance, energy and a greater degree of resolution than we observe in the average working classes. The mitral valve giving way to a greater or less extent under pressure, the patient becomes more embarrassed for breathing; it fails to close, letting the blood regurgitate back upon the lung at every stroke. "Damming" the blood in the lungs again has the effect of impeding the right side to some extent; the auriculo-ventricular opening dilates and renders the tricuspid incapable of closing; thus you get a pulmonary regurgitant condition on that side. With this embarrassment, giving inadequate force to the circulation in the distant parts of the body, there develops within a few weeks general anasarca. The patient begins to be filled up with effusion, and simultaneously effusion takes place, more or less, into the pericardium; so now we have the pericardium distended to such an extent as to increase largely the cardiac space of dullness.

Another point: When you get the sounds of the heart I have mentioned, you know where to find the regular cardiac impulse. When you place your stethoscope on either of the areas named, you fail to get an impression of the impulse, because the heart does not strike the walls of the chest in its natural manner; you get a sound as though it was removed from the walls of the chest to a little distance. Where you get a distinct sound and absence of impulse, with an increased area of dullness, you get indications of pericardial effusion, and the pericardial effusion keeps the intercostal spaces more or less filled up.

I think the liver also, in this state of circulation, has become somewhat enlarged from engorgement, giving more dullness than would otherwise arise there; although if the area of dullness were on that side, it might depend partly on effusion into the pleuræ as well as into the pericardium. We get no acknowledgment of inflammation of either the pericardium or pleuræ, or any sharp pains anywhere. I suppose the effusions into the pericardium and pleuræ, if they exist, are identical with the effusion into the legs. It is possible the effusion results from an inadequate circulation and an impoverished condition of the blood, and if so, the tendency would be to increase and fill up the parts quite rapidly. To explain the imperfect inflation of the lungs, and the sharp, crackling sound with each inspiratory act, is not an easy matter. There is no expectoration indicating a pneumonic condition; no cough, such as we would expect from a general dry bronchitis. I apprehend that this imperfect inflation of the lungs results from an increased space, occupied by the heart and its membranes, and enlargement of the liver.

It would add much to the interest of the case if we could get an accurate, reliable history, but under the circumstances we must endeavor to meet the indications.

What can be done for such a patient? Remember the œdematous condition of the legs and the indications of effusion. The question might arise in your mind whether this man has not had some disordered condition of the kidneys, or albuminuria, for during the progress of albuminuria we get the development of cardiac attacks in some form; sometimes the accentuation of the second sound of the heart over the aortic cartilage, and reduplication of the first sound over the septum ventriculorum, inflammatory actions, effusions, etc. The heart becomes more or less enfeebled in the progress of that affection. This man, however, has not the look of an albuminuria patient. Albuminuria gives your patient a decided paleness, dryness of the skin, whiteness of the surface; and then, so far as we are able to ascertain, there is little or no albumen in this patient's urine—in short, nothing special about him to direct our attention to the kidneys or urinary secretions.

If we work on the theory or principle that cardiac weakness in this, as in many other cases, is caused by the failure of oxygenation and decarbonization of the blood, anything that will improve the condition of our patient, so the object is to use such remedies—together with good nourishment, plenty of fresh air, keeping the patient in as easy and comfortable position as possible—as will aid the circulation, increase, if possible, the systolic muscular force of the heart. I have for a great many years relied upon digitalis. Where you can get the remedy adjusted as to frequency and size of the dose, so as to get the heart-beats down between 67 and 70 in a minute, you almost invariably give relief to your patient. The most careful experimentation bearing upon the physiological action of this drug shows that it acts on the vaso-motor nerve centres, increases the action of the muscular coats of the vessels, contracts the arterioles, stimulates the motor ganglia in small doses, increasing the tone of the vascular system throughout, at the same time lessening the frequency of the heart's beat. We desire simply to slow the heart's beat, to give more time between the beats for the blood to fill up the respective cavities, to sustain the heart and not increase the tone of the vascular system throughout to any great extent. It is maintained that digitalis, on account of increasing the tone of the vascular system, increases also the resistance of the circulation through the capillaries. I have not found evidences of embarrassment in that direction in using the drug.

Recently the remedy known as *strophanthus* has been thoroughly investigated and brought into use, the drug producing all the beneficial effects that digitalis does, rendering the heart-beats slower, sustaining its muscular force without increasing the tension of the vascular system, consequently it is claimed that it meets all the indications of digitalis in the treatment of cardiac affections.

In this case we need have no fear of increasing too much the tone of the vascular system. There is a lack of vaso-motor influence on the part of the vascular system as well as enfeeblement of the heart as the central organ of circulation.

For many years, in such cases as this, my favorite prescription has been the tincture of digitalis and fluid extract of scutellaria.

Scutellaria is classed in your materia medica, I believe, as a nervine tonic. The idea is maintained that its effects are somewhat quieting to the nervous system, and that it is a mild tonic. My experience is that it does more than that, but yet I am not prepared to explain the *modus operandi* of it fully. I give it along with digitalis on account of its mild tonic influence, improving to some extent the digestion, increasing the tone of the stomach. I use it a great deal, in addition to other remedial agents, in treating dyspepsias, various forms of indigestion arising from functional disturbances of the stomach—two parts of the extract of scutellaria and one part tr. digitalis.

I would recommend in this case, to commence with, 10 drops fluid ex. scutellaria and 5 of digitalis, to be given every four hours. If, after 24 hours, there is no perceptible change in the condition of the pulse, I would increase the dose to 18 drops, or thereabouts; let it go on another 24 hours and watch the result. If still the pulse ranged about the same, I would add 2 drops more; indeed, I should expect but little physiological influence until I reached 20 minims at a dose of this mixture. You seldom, as a rule, have to give more than a few doses before you find the pulse diminished to its natural standard and usually a little below. You will perhaps find it between 60 and 70 beats in a minute, with an increase of the systolic force, there being now a good, full interval between, and, at the same time, we generally find the respiration correspondingly improved, and the general aspect of the patient in every way better. When you succeed in getting the pulse slightly below normal—say 60 to 70 beats in a minute—you should begin immediately to lengthen the interval between doses, or diminish their size, so as not to allow the pulse to go beyond its natural beat, if possible—keep it just normal, or slightly below, and if you do that it will maintain the circulation and stimulate the condition of your patient more than anything I know.

Strophanthus I have been using for the last six months, and while I cannot speak definitely of its merits and good qualities at this time, I am inclined to think that it is a good remedy, and will undoubtedly supersede digitalis in many cases in which we are using it. It is still on trial.

